# Social self-efficacy and mental well-being in autistic adults: Exploring the role of social identity

**DOI:** 10.1177/13623613231195799

**Published:** 2023-09-20

**Authors:** Lorna Camus, Gnanathusharan Rajendran, Mary Elizabeth Stewart

**Affiliations:** Heriot-Watt University, UK

**Keywords:** adults, autism, double empathy, homophily, mental well-being, social identity, social self-efficacy

## Abstract

The double empathy problem proposes a lack of shared understanding between autistic and non-autistic people leads to interaction difficulties between them. Social self-efficacy (confidence in one’s social abilities) makes an important contribution to social interactions. Research has shown that autistic people show a social identity (one’s sense of self based on membership of social groups) with other autistic people, and this impacts positively on well-being. In addition, research shows that autistic people have a number of social identities. This study aims to understand whether social self-efficacy relates to mental well-being in autistic adults, and whether social identity plays a role in this relationship. A total of 512 autistic adults completed measures of social self-efficacy, social identity, mental well-being and autistic traits. In-group social self-efficacy was found to be higher than out-group social self-efficacy. Second, in-group social self-efficacy was positively associated with well-being. Finally, social identity did not mediate the relationships between social self-efficacy and mental well-being. These findings suggest social self-efficacy may relate to the nature of the group one interacts with. The findings support further research into the relationship between social self-efficacy and well-being, as well as using the double empathy problem as a framework for further investigations in other social groups.

**Lay abstract:**

In the past, research has suggested that autistic people are not able to communicate well with non-autistic people because of autistic people’s communication difficulties. However, newer theories question this conclusion. It is now thought that the communication difficulties may be because autistic and non-autistic people both struggle to understand each other. This study explores how these differences in shared understanding relate to autistic people’s mental well-being, confidence in social situations and social identities (groups that we belong to and that influence how we see ourselves). We created an online survey taken by 512 autistic adults, which included questions about their confidence being social with people from different groups (such as other autistic people or people they share a hobby with), about the social groups they felt they belong to and about their mental well-being. First, participants reported higher social confidence when interacting with members of a social group they belonged to. Second, being confident during these interactions was linked to higher mental well-being. Finally, the groups participants belonged to did not influence the link between social confidence and mental well-being. These findings are important as they help us better understand autistic people’s experiences of social interactions and what contributes to good and poor mental well-being in autistic people. They also help us to think further about how to improve autistic people’s well-being.

## Introduction

A growing body of research now explores autistic interactions, putting in question past understandings of social interaction deficits in autism. The double empathy problem (Milton, 2012) proposes that a lack of *shared* understanding between autistic and non-autistic people leads to communication difficulties, questioning the idea that social interaction difficulties are due to issues with autistic people’s communication. Milton (2012) suggests that autistic and non-autistic people’s experiences and perceptions of the world are mismatched, which leads to difficulties for both to understand and communicate with each other. While empathy (or lack thereof) is a factor in this theory, Milton’s double empathy problem suggests a more general issue regarding autistic and non-autistic people lacking insight into the other group’s thoughts and culture (including societal norms). In this sense, the double empathy problem could be seen as being related to the construct of homophily (the tendency to bond and relate with those similar to oneself, seen in autistic and non-autistic people; Farmer & Farmer, 1996; Gernsbacher et al., 2017; Koutsouris, 2014). Both the double empathy problem and the construct of homophily suggest that people who are more similar tend to bond and relate to each other. The double empathy problem also incorporates the role that societal norms (such as expecting eye-contact or other ‘normal’ social behaviours in interactions) and the othering of difference (such as with making autistic people feel different to others due to differences in communication styles) play in perpetuating misunderstandings between autistic and non-autistic people (and in labelling autistic people as deficient or abnormal).

Qualitative studies have found autistic people report that interactions with other autistic people are easier and lead to less misunderstandings (Crompton, Hallett, et al., 2020) which may improve the quality of, intimacy within and satisfaction with a given relationship. In addition, interaction flow, rapport and attunement are significantly increased in autistic interactions compared to autistic/non-autistic (mixed) interactions (Williams et al., 2021). Similarly, quantitative studies testing the double empathy problem empirically have found that autistic interactions are higher in rapport than mixed interactions (Crompton, Ropar, et al., 2020; Crompton, Sharp, et al., 2020). Morrison et al. (2020) also found autistic people disclosed more information about themselves with other autistic people. In a diffusion chain experiment (in which participants must pass information along a chain to the next participant), Crompton, Ropar, et al. (2020) found that pairs of autistic participants were equally successful in sharing information as compared to pairs of non-autistic participants, while mixed pairs shared significantly less information. These findings suggest that autistic people may have a different experience and, in some respects, can be more successful (e.g. higher information transfer) in interactions with other autistic people.

A potential avenue of interest regarding our understanding of social interaction and similarity to others is to extend this to groups outside of autistic and non-autistic people. A person’s social identity is built upon their perception of belonging to certain social groups. It is therefore part of their self-concept, influencing how they think about themselves relative to members of the same or of different groups. Indeed, social identity theory (Tajfel et al., 1979) suggests a person’s social identity includes attributes which identifies them (as perceived by themselves and other people) as similar to members of groups they belong to (the in-group) and different to members of groups they do not belong to (the out-group). For example, a person may identify with friends, family or members of a hobby group. The theory then considers how this process influences intergroup behaviours, such as in-group favouritism (favouring members of an in-group and discriminating against members of an out-group). By taking into account social identity theory, one might hypothesise there is a shared understanding between members of the same social group.

Both the theories of homophily and the double empathy problem highlight that similarities between groups may result in more positive interactions. It may therefore be helpful to explore these within the context of other social identities as members of the same social group may also perceive the world more similarly to each other (and therefore experience more positive interactions with their in-group and interactional difficulties with their out-group).

Social self-efficacy is a person’s confidence in their ability to successfully establish and sustain social interactions and friendships (Gecas, 1989). It can make an important contribution to social interactions by mediating the association between relational factors (such as attachment anxiety and avoidance) and perceptions of social support (Mallinckrodt & Wei, 2005) and loneliness (Wei et al., 2005). Extant research shows that social self-efficacy is important to both a wide range of other interpersonal factors and to outcomes and performance (Dunbar et al., 2018; Raskauskas et al., 2015; Wei et al., 2005). There are several studies which have assessed social self-efficacy or social competence with autistic people or assessed relationships with autistic traits. For example, in a sample of 22 autistic children, self-evaluated social competence was lower compared to a non-autistic sample mean (Vickerstaff et al., 2007), and self-rather than other-rated social ability was related to depression symptoms. Rosbrook and Whittingham (2010) found that self-rated social competence was negatively correlated with autistic traits in a sample of 231 university students.

Given the importance of the social self, it seems reasonable to expect there to be a relationship between social self-efficacy and social identity. Despite limited research in this area, there are some studies which explore this relationship. For instance, Guan and So (2016) found self-efficacy of engaging in health-related behaviours promoted by a group was higher for individuals who identified more strongly with this group. Similarly, Gushue et al. (2006) found that higher vocational self-efficacy was positively associated with vocational identity in their sample of high school students. In a study of entrepreneurial self-efficacy, Brändle et al. (2018) found different types of entrepreneur identities were associated with different levels of self-efficacy. So, in the context of the theories of social identity, double empathy and homophily, social self-efficacy may be dependent on an individual’s social identities, where interacting with a member of a group one belongs to relates to higher social self-efficacy.

Taking into account these theories, and despite the currently limited evidence, it may be possible that autistic people’s mental well-being is affected by a complex combination of social self-efficacy, the social identities they belong to and miscommunication with non-autistic people – as captured by the double empathy problem. Autistic people are more likely to experience mental health difficulties at some point in their lives, especially depression and anxiety (Lever & Geurts, 2016; Simonoff et al., 2008). It is likely a complex set of interacting factors contribute to this, including difficulties associated with being autistic and unaccommodating environments.

Research exploring the role of double empathy, and specifically the experience of miscommunication and misunderstandings between autistic and non-autistic people, on autistic mental well-being is in its infancy. However, recent theoretical work by Mitchell et al. (2021) has proposed a link between double empathy and mental well-being, which is supported by Camus, Macmillan, et al. (2022). Mitchell et al. propose that double empathy, exemplified by the non-autistic majority systematically misunderstanding autistic people, can result in autistic people being excluded from social life. This itself can negatively affect mental well-being. In turn, this can lead to autistic people feeling the need to camouflage, which has been shown to be associated to poorer mental health outcomes for this population (Bargiela et al., 2016; Cassidy et al., 2018). Camus et al. support this proposed association between double empathy and mental health difficulties, reporting the experience of autistic adults with double empathy misunderstandings and their negative impact on their lives and mental health.

Social self-efficacy may also be related to mental well-being in autistic people. A cross-sectional study in a predominantly non-autistic sample found the relationship between autistic traits and depressive symptoms to be serially mediated by social self-efficacy, social motivation and loneliness (in that order) (Camus, Jones, et al., 2022). This study suggests that targeting social self-efficacy may disrupt this pathway and improve mental health outcomes in those with high autistic traits. Previous research supports these findings, with studies finding lower social self-efficacy in samples of autistic participants (Vickerstaff et al., 2007) and those with high autistic traits (Rosbrook & Whittingham, 2010) and finding a relationship between social self-efficacy and depressive symptoms (Vickerstaff et al., 2007; Wei et al., 2005). Therefore, the relationship between social self-efficacy and mental well-being in autistic people should be examined to assess whether social self-efficacy has an impact on autistic people’s well-being.

The aim of the study is to understand whether social self-efficacy relates to mental well-being in autistic adults, and whether social identity plays a role in this relationship. It was predicted that in-group social self-efficacy would be higher than out-group social self-efficacy and that higher in-group social self-efficacy would be positively related to mental well-being. In addition, we hypothesised that participants’ number of social identities would mediate this relationship.

## Methods

### Public involvement

In order to assess the study’s applicability and acceptability with the autistic community, the first author created an online visual presentation outlining this study’s topic, methods and expected results and their dissemination, which was distributed via the first author’s Twitter account. Three autistic adults and three autistic parents of autistic children were also invited to provide insight and feedback on the study’s topic, methods and materials via the Autistica Insight Group. Feedback suggested that generally, the autistic community considered the planned study to be worthwhile. Some changes to the original methods were made following feedback, including raising the starting age of the sample from 9 to 12 years old, including an explicit explanation of how the study’s results would be used in both the information and debrief documents shared with participants and adding other social groups to the social identity questions. We thank all those who provided feedback and hope that consequently, this study offers a greater contribution to the autistic community.

### Participants

Five hundred twelve out of 666 participants were included in data analyses. Complete cases for the different measures ranged from 435 to 512. A total of 89 participants were excluded as they were missing data for 2 or more measures, and 11 were excluded as they were outliers. In addition, the child sample was too small to achieve power (*N* = 54) and was therefore excluded from the analyses. The sample was aged between 16 and 88 years old (M = 42 years old). Recruitment was targeted to include autistic and non-autistic participants. Participants were recruited via social media, mailing lists, the Autistica Discover Network, the Scottish Women’s Autism Network (SWAN), the Stirling Autism Research group, the Scottish Autism Research Group (SARG), the Cambridge Autism Research Database and the research website Call For Participants. We included 16- and 17-year-olds as the Mental Capacity Act 2005 defines an adult as a person aged 16 years or older. A total of 290 participants were women, 159 men, 33 identified as non-binary, 9 identified as transgender (male or female), 17 self-described in other terms and 4 did not answer. The majority of participants identified as White (414 participants). Finally, most participants had been formally diagnosed as autistic (397), while 115 participants self-identified as autistic.

### Measures

#### Social self-efficacy

The Social Self-Efficacy Scale (SSES, Grieve et al., 2014) was used twice to measure participants’ perception of their ability to socialise first with members of their in-group and then with members of an out-group. Participants are asked to rate how confident they are in 18 aspects of social interactions (e.g. ‘enter new situations and meeting people for the first time’). Ratings are made on a 5-point scale ranging from ‘not at all confident’ to ‘very confident’, resulting in scores ranging from 18 to 90. The SSES has shown good psychometrics in previous studies (including very high internal consistency with α = 0.94, Grieve et al., 2014). Internal consistency of the SSES in this study was very high for both the in-group and the out-group measures (α = 0.952 and α = 0.956).

#### Social identity

Social identity was measured using an abbreviated version of Leach et al.’s (2008) 14-item measure of social identification. To lessen the burden on participants, only 2 of the 14 items were chosen to assess group identification and provide context for participants’ answers to the SSESs. As the SSES was completed once for an in-group and once for an out-group, the social identity questions provided participants with groups to reference when completing the social self-efficacy questions. The first item chosen was taken from the Solidarity component (‘I feel a bond with (group)’). It was chosen as the Solidarity component represents a person’s sense of belonging and psychological attachment to the group, which is central to a person’s social identification with a group (Leach et al., 2008). The second item chosen was taken from the Centrality component (‘Being part of (group) is an important part of how I see myself’). This item was chosen as this component reflects the salience and importance of group membership, which makes a group central to how a person perceives themselves.

For each question, participants rated their agreement on a 7-point scale (1 = strongly disagree, 7 = strongly agree) in relation to seven different groups (autistic people, family, significant other, classmates, co-workers, hobby group, volunteering group). Overall scores for each question ranged from 7 to 49. The seven groups were chosen based on consultations with autistic people, who suggested using specific groups (such as co-workers or volunteering group) was clearer and less abstract. Internal consistency for each question was excellent (α = 0.877 and α = 0.931 respectively).

Social identity was used in analyses in two ways. First, as a total number of identities adhered to (ranging from 0 to 7). Scores over 4 on both questions for each individual group were considered to indicate a social identity (i.e. participants must score over 4 on both questions for a group to be considered a social identity). If data were not provided for a group, the group was not considered to be a social identity for that individual. Therefore, a participant’s number of social identities is the number of groups for which their average social identification score equalled 4 or more.

Second, identities were split into non-optional and optional social identities. Non-optional identities are those the participant does not actively seek out, such as being autistic, or being part of a family, or group of co-workers. Optional identities are those the participant will have sought out, such as being part of a hobby or volunteering group. Both these variables were dichotomised, so that participants were noted as either having a non-optional/optional identity or not having one (regardless of how many of these non-optional or optional identities they had). Participants with one or more optional identities were given a score of 1 on the optional identity variable, and 0 if they had no optional identities. The same was done for non-optional identities.

#### Mental well-being

Mental well-being was measured using the Short Warwick-Edinburgh Mental Well-being Scale (SWEMWBS, Stewart-Brown et al., 2009), a 7-item self-reported scale. The short scale includes items relating to functioning more so than feelings. Participants are asked to report their experiences over the past 2 weeks and to rate items on a 5-point scale ranging from ‘none of the time’ to ‘all of the time’. This results in a total raw score ranging from 7 to 35, which is then transformed into a metric score using the SWEMWBS conversion table (also ranging from 7 to 35). Higher scores indicate higher positive mental well-being. The SWEMBWS has good psychometric properties (McKay & Andretta, 2017; Vaingankar et al., 2017). Internal consistency of the scale was excellent in this study (α = 0.831).

#### Autistic traits

A 10-item, self-report scale, the Autism-Spectrum Quotient–10 items (both the Child and Adult versions) (Allison et al., 2012), was used to measure autistic traits in children and adults respectively. Items were scored using the recommended dichotomous scoring system creating scores ranging from 1 to 10, with scores of 6 and above considered to reflect high autistic traits. The total Autism Spectrum Quotient (AQ) score was used in analyses. Psychometrics for the AQ-10 (Child and Adult versions) were found to be satisfactory (Allison et al., 2012). Internal consistency of the AQ scores in this study was moderate for both the Adult and Child versions (α = 0.613 and α = 0.671, respectively).

### Procedure

Participants were recruited to complete questionnaires in an online survey hosted on Qualtrics (2017). Information on the study was provided, and participants were required to consent to participate prior to starting to complete the questionnaires. Participants were also informed that upon completing the study they would be entered into a prize draw to win one £50 voucher or one of four £40 vouchers. The project was granted ethical approval by University Ethics Committees. The survey collected demographic information (gender, age, ethnicity) and medical information (autism diagnosis) as well as the outlined questionnaires. The questionnaires pack took on average 21 minutes to complete. Once data collection was completed, data were coded and anonymised before being analysed in R (R Core Team, 2019).

### Analysis

The difference in social self-efficacy for in-group and out-group members was examined via a *t*-test. The relationship between in-group social self-efficacy and mental well-being was assessed via a hierarchical regression using two models. The first model predicted mental well-being from age alone, while the second model added in-group social self-efficacy as a predictor, controlling for age. Finally, to assess whether the number of social identities mediates the relationship between social self-efficacy and mental well-being, two mediation analyses were carried out (for in-group and out-group social self-efficacy).

**Figure 1.**
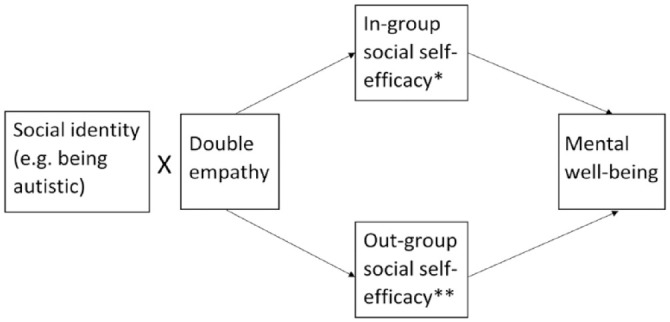
Proposed relationships between social identity, double empathy, social self-efficacy and mental well-being. *For example, social self-efficacy related to interactions with other autistic people. **For example, social self-efficacy related to interaction with non-autistic people.

The proposed relationships between social identity, double empathy, social self-efficacy and mental well-being are illustrated in Figure 1.

## Results

### Descriptives

There were 42 cases of multivariate outliers (participants with a combination of unusual scores on at least two variables). However, upon individual inspection, 31 outliers were coherent within their own scores and within the range of scores of the sample; therefore, they were not excluded. A total of 11 outliers were not coherent, with inconsistent social self-efficacy scores and social identity scores (e.g. reporting no social identities but scoring high on social self-efficacy) and were therefore excluded from the rest of the analyses.

Analyses revealed there were no significant gender differences for well-being, *t*(326) = −2, *p* = 0.08; number of social identities *t*(284) = −0.2, *p* = 0.8; in-group social self-efficacy *t*(248) = 0.5, *p* = 0.6; and out-group social self-efficacy scores, *t*(293) = −1, *p* = 0.3. As no gender differences were found for any of the measures, gender was not included in the rest of the analyses.

Descriptive statistics for the sample are reported in Table 1.

**Table 1.** Descriptive statistics.

	*n*	Mean	SD	Minimum	Maximum
AQ	495	7.57	1.99	1.00	10
Well-being	498	18.69	3.42	9.51	35
In-group SSE	435	53.55	17.39	21.00	105
Out-group SSE	491	41.20	16.03	20.00	105
Number of social identities	512	2.19	1.65	0.00	7

AQ: Autism Spectrum Quotient; SSE: social self-efficacy.

Table 2 reports the social identities of participants. Participants may adhere to more than one identity.

**Table 2.** Social identities of participants.

Groups	*N*	Is an identity	Is not an identity
Autistic people	344	247	97
Family	395	235	160
Significant other	281	217	64
Co-workers	251	113	138
Classmates	177	46	131
Hobby group	220	148	72
Volunteering group	171	113	58

**Figure 2.**
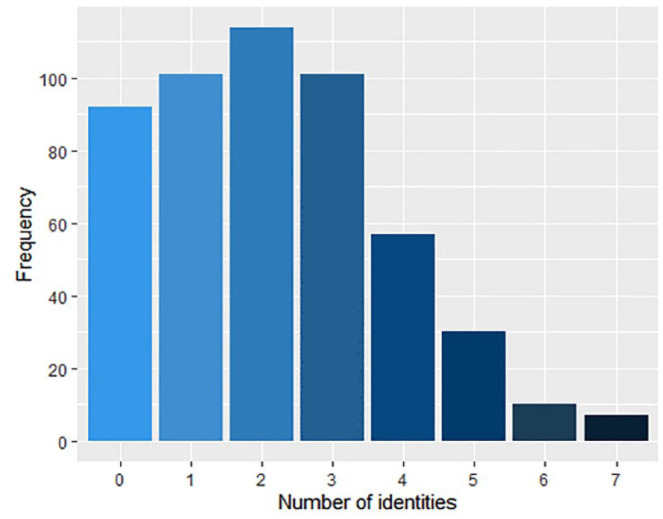
Frequency of reporting 0–7 social identities.

Figure 2 illustrates frequencies among the sample of identifying with 0–7 social identity groups.

### *t*-Test

A *t*-test was conducted in order to assess whether in-group social self-efficacy was higher than out-group social self-efficacy.

As hypothesised, participants reported higher in-group social self-efficacy (M = 53.55, SD = 17.39) than out-group social self-efficacy (M = 41.20, SD = 16.03), *t*(413) = 19, *p* < 0.001.

### Regressions

As mental well-being was related to all other measures of interest (Table 3), we then examined the relationship between social self-efficacy and well-being. This relationship was assessed using a hierarchical regression with two models. The first model used only age to predict mental well-being, while the second model added in-group social self-efficacy as a predictor, while controlling for age.

**Table 3.** Correlation matrix.

	Age	Mental well-being	In-group SSE	Out-group SSE	Number of SIs	Non-optional SI	Optional SI
Age	–						
Mental well-being	0.076	–					
In-group SSE	−0.337***	0.377***	–				
Out-group SSE	−0.230***	0.332***	0.687***	–			
Number of social identities	−0.177***	0.324***	0.335***	0.215***	–		
Non-optional SI	−0.115*	0.181***	0.06	−0.036	0.625***	–	
Optional SI	−0.085	0.217***	0.240***	0.195***	0.627***	0.247***	–

SI: social identity; non-optional SI: having 1 or more non-optional social identities; optional SI: having 1 or more optional social identities; SSE: social self-efficacy.

* *p* < 0.05; ***p < 0.001.

The first regression model, including only age, was not significant (*p* = 0.09) and accounted for less than 1% of the variance in mental well-being (*R*^2^ = 0.009). The final regression model, adding in-group social self-efficacy, was statistically significant (*p* < 0.001) and accounted for 24% of the variance in mental well-being (*R*^2^ = 0.235).

As predicted, in-group social self-efficacy had a significant positive effect on well-being scores (β = 0.10, *t*(281) = 9.20, *p* < 0.001), even after controlling for age (β = 0.07, *t*(281) = 5.17, *p* < 0.001). As age and in-group social self-efficacy increase, so does mental well-being.

### Mediation analysis

Finally, to assess whether the number of social identities one has mediated the relationship between social self-efficacy and mental well-being, two mediation analyses were carried out (one for in-group social self-efficacy and one for out-group).

**Figure 3.**
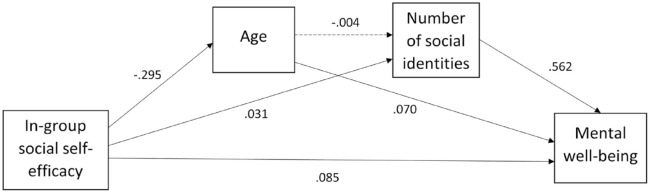
Mediation model of in-group social self-efficacy as a predictor of mental well-being, mediated by number of social identities and controlling for age. Dashed pathways are non-significant. Numbers refer to pathway estimates.

Contrary to our hypotheses, the effect between in-group social self-efficacy and mental well-being was not mediated by the number of social identities participants had. The indirect pathway of the effect of in-group social self-efficacy on mental well-being via age and number of social identities was not significant (β (indirect) = 0.001, *z* = 0.745, *p* = 0.456). This pathway did not account for the overall impact of in-group social self-efficacy on well-being as this direct effect was significant (β (direct) = 0.085, *z* = 7.445, *p* < 0.001). Figure 3 illustrates these pathways.

**Figure 4.**
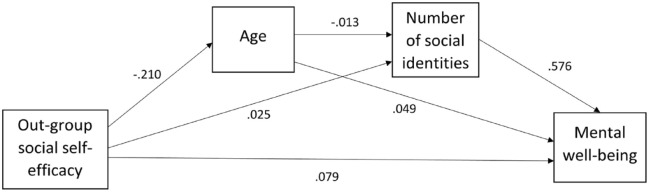
Mediation model of out-group social self-efficacy as a predictor of mental well-being, mediated by number of social identities and controlling for age. Numbers refer to pathway estimates.

Similarly, the effect between out-group social self-efficacy and mental well-being was not mediated by the number of social identities participants had (see Figure 4). The indirect pathway of the effect of out-group social self-efficacy on mental well-being via age and number of social identities was not significant (β (indirect) = 0.002, *z* = 1.851, *p* = 0.064). This pathway did not account for the overall impact of out-group social self-efficacy on well-being with the direct effect being significant (β (direct) = 0.079, *z* = 7.157, *p* < 0.001).

These relationships did not change when looking at the mediating role of optional versus non-optional social identities (as opposed to number of social identities).

## Discussion

The aim of this study was to assess whether social self-efficacy relates to mental well-being in autistic adults, and whether social identity plays a role in this relationship. As expected, social self-efficacy was positively related to well-being in this sample of autistic adults, which is in line with the previous research finding that social self-efficacy mediates the relationship between autistic traits and depressive symptoms (Camus, Jones, et al., 2022). In line with our predictions, the sample’s out-group social self-efficacy was lower than for their in-group. Within the context of the social identity theory, these findings suggest social identity may play a role in how confident autistic people can be when interacting with others, and in turn, this may have an impact on their mental well-being.

These findings support and extend the research by Maitland et al. (2021) and Cooper et al. (2017). Cooper et al. found that autistic people’s self-esteem was associated with how much they identified with other autistic people, which in turn affected their mental health. Maitland et al. (2021) found that stronger feelings of social identity and the number of groups identified with were associated with more positive mental health (well-being and fewer depressive symptoms) in autistic people. The strength of social identity and number of social identities has robustly been found to be related to positive mental health (Sani et al., 2012). Similarly, we found that both in- and out-group social self-efficacy was correlated with mental well-being and that the number of social identities was correlated with all three variables. Of particular interest is that although both types of social identity (optional and non-optional) were correlated with mental well-being, social self-efficacy was only correlated with optional social identity. However, neither number and/or type of social identities mediated the relationship between social self-efficacy and mental well-being.

The findings may be interpreted within either the double empathy problem or the homophily theory which both suggest that people who are similar (such as those belonging to the same social groups) find it easier to bond with and understand each other. This may therefore have an impact on how confident a person will be interacting with a member of their in-group or members of out-groups, as seen in this study, thereby supporting the rationale for continuing to explore these theories in relation to autism and other groups.

This study’s results suggest that targeting social self-efficacy for intervention may help support autistic people’s well-being. Social self-efficacy was found to be higher for participants’ in-groups; therefore, a potential avenue for intervention would be to support widening autistic people’s in-groups via the creation and support of social groups. It is well known that interventions such as the development of peer support can support positive well-being, such as helping with reducing depressive symptoms (Pfeiffer et al., 2011) or with post-diagnosis support for newly diagnosed autistic adults (Crompton, Hallett, McAuliffe et al., 2022). Recent studies exploring the attitudes of autistic people towards such peer support interventions have shown these types of interventions are desirable and a potentially beneficial alternative to more traditional forms of support for this population (Crompton, Hallett, Axbey et al., 2022; Valderrama et al., 2023). Future research could therefore develop autistic peer support interventions and assess whether they have a positive effect on autistic people’s mental well-being (Crompton, Hallett, et al., 2020).

Moreover, our findings suggest further exploration into the double empathy and homophily theories, as well as their role in different social groups. If more studies support double empathy (specifically in the context of interactional difficulties such as misunderstandings and miscommunication) having an effect on autistic people’s mental well-being, this would suggest avenues for interventions, such as increasing autistic interactions or increasing shared understanding between autistic and non-autistic people. However, as autistic people represent a small part of the general population, interventions based on increasing autistic interactions may not be sufficient to improve autistic people’s well-being. This may be resolved by extending the double empathy problem to other social groups as done in this study. Interventions involving the creation or support of social groups may therefore extend to other social groups autistic people identify with, over and above autistic groups.

This study has several limitations. First, replication of these findings in longitudinal and experimental designs is needed to better understand the direction of the relationships found in this study. Second, the social identity measure used in this study was an abbreviated version of a validated measure. While the measure’s internal consistency for this study was very high, replication with the full measure would be of interest. Moreover, this study does not reflect the unique experience of individuals who are members of multiple social minority groups. Indeed, the quality of the connection to these multiple groups may be poorer as individuals may have trouble identifying with any group fully and identifying to multiple minority groups may compound experiences of stigma or discrimination. While many autistic people identify strongly with autism as a social identity (Cooper et al., 2021), which can have a protective effect for their mental health (Cooper et al., 2023), those with additional minority identities may struggle identifying with these groups and may suffer greater discrimination (McAuliffe et al., 2022; Sedgewick et al., 2020). The study did not include any assessment of how salient the social groups where for the autistic participants nor the quality of the interaction, factors which may affect level of identification, reports of membership and impact on mental health.

Future research should aim to delve deeper into the relationships between social self-efficacy and social group identification to examine this experience. Similarly, the number of participants identifying as non-binary, transgender or otherwise genderfluid was too low to compare with cisgender male and female participants. As autistic people are more likely to identify as non-binary, transgender or otherwise genderfluid (Janssen et al., 2016) and those who do may experience higher levels of mental health difficulties (Sedgewick et al., 2020), it will be important to replicate these findings in a sample consisting of more non-binary or transgender people. It would be interesting for future research to explore how supporting in-group interactions and expanding a person’s number of in-groups may support mental well-being.

As this study’s results can be interpreted through the lens of the double empathy problem future research could also try to test this in non-autistic samples to determine whether the findings of this study also apply to non-autistic people.

## Conclusion

In conclusion, this study supports further exploring the theories of double empathy, homophily and social identity as they relate to autism, mental well-being and different social groups and factors such as social self-efficacy. It also grants new insights into applications of the double empathy problem to other social groups by accounting for social identity theory. Our results indicate targeting social confidence may provide mental health benefits in autistic people, and further research is needed to determine how this can be implemented. While the number or type of social identities did not mediate this relationship in this study, replication with a complete measure of social identity is needed. Finally, it would be insightful for future research to examine how supporting in-group interactions supports autistic people’s mental well-being, and whether belonging to more social groups can improve an individual’s mental well-being.
